# Zirconium Component Modified Porous Nanowood for Efficient Removal of Phosphate from Aqueous Solutions

**DOI:** 10.3390/nano13111807

**Published:** 2023-06-05

**Authors:** Zhuangzhuang Chu, Wei Wang, Mengping Yin, Zhuohong Yang

**Affiliations:** 1 Key Laboratory for Biobased Materials and Energy of Ministry of Education, College of Materials and Energy, South China Agricultural University, Guangzhou 510642, China; zhuangc@scau.edu.cn (Z.C.); 18186614451@163.com (W.W.); sadheud@163.com (M.Y.); 2 College of Natural Resources and Environment, South China Agricultural University, Guangzhou 510642, China

**Keywords:** natural wood, zirconium, nanocomposite, phosphate, adsorption

## Abstract

Rapid urban industrialization and agricultural production have led to the discharge of excessive phosphate into aquatic systems, resulting in a rise in water pollution. Therefore, there is an urgent need to explore efficient phosphate removal technologies. Herein, a novel phosphate capture nanocomposite (PEI−PW@Zr) with mild preparation conditions, environmental friendliness, recyclability, and high efficiency has been developed by modifying aminated nanowood with a zirconium (Zr) component. The Zr component imparts the ability to capture phosphate to the PEI−PW@Zr, while the porous structure provides a mass transfer channel, resulting in excellent adsorption efficiency. Additionally, the nanocomposite maintains more than 80% phosphate adsorption efficiency even after ten adsorption–desorption cycles, indicating its recyclability and potential for repeated use. This compressible nanocomposite provides novel insights into the design of efficient phosphate removal cleaners and offers potential approaches for the functionalization of biomass−based composites.

## 1. Introduction

Phosphorus fertilizer is a crucial component of chemical fertilizers in agricultural production, facilitating plant resistance and growth [1]. However, high levels of phosphorus in the environment can have adverse effects [2,3]. For instance, the excessive use in agriculture can cause phosphorus to leach into surface water [4], while the use of domestic and industrial detergents can result in excess phosphate in wastewater, which can end up in water bodies [5]. A significant concern is when the phosphorus concentration in water bodies exceeds 0.01–0.02 mg·L^−1^, leading to toxic algal growth, overgrowth, and eutrophication [6]. Hence, it is critical to remove phosphorus from natural water bodies, particularly freshwater bodies, to prevent eutrophication.

Phosphorus exists in pentavalent forms such as orthophosphate, pyrophosphate, long−chain polyphosphate, and phosphodiester in the aqueous environment [7]. Figure S1 presents the structural formulas for these forms of phosphorus. These different phosphorus−containing compounds can be hydrolyzed to orthophosphate, which is the only form that bacteria, algae, and plants can utilize [8]. Consequently, it is crucial to remove excess orthophosphate from water bodies [9,10]. Various methods for the removal of phosphate exist currently, including biological treatment [11], chemical co−precipitation [12], reverse osmosis [13], electrodialysis [14], membrane separation [15], and adsorption [16]. Among these, the adsorption method is widely used in the field of wastewater treatment because of its high efficiency, environmental friendliness, recyclability, and flexibility in preparation and separation, making it a promising approach for phosphorus capture in wastewater [17].

Several adsorbent materials have been explored for phosphate removal from water, including biochar [18], laponite [19], and biopolymers [20,21]. Despite the progress made, many prepared adsorbents exhibit drawbacks such as high cost, non−recyclability, poor adsorption capacity, and low adsorption rate, thereby limiting their practical use [22]. Wood, as a renewable and carbon−neutral resource, has emerged as an attractive raw material in the field of advanced materials [23,24]. Wood possesses numerous advantages, such as large specific surface area, regular pore channels, biodegradability, non−toxicity, and diverse tunability through functional groups such as hydroxyl groups [25]. However, wood has poor adsorption performance for phosphates due to the abundance of electronegative functional groups, which hinder the adsorption of anions [26]. Therefore, modifying wood−based materials is necessary to achieve effective phosphate adsorption.

Recently, considerable research efforts have been focused on developing novel adsorbent materials modified with phosphate−affinity components, such as lanthanum or Zr, to enhance their adsorption capacity for phosphate ions [27]. Among these, Zr−based oxides have been identified as a promising adsorbent for phosphate removal due to their superior properties, including high thermal stability, low solubility in water, and resistance to oxidants and acids/bases [28,29]. Zr components not only endow adsorbents with ultra−high adsorption affinity for phosphate, but also impart selectivity towards phosphate ions in water [30,31]. As such, incorporation of Zr components into modified wood−based materials offers a potential approach for the efficient removal of phosphate from wastewater.

In this study, a novel adsorbent material for phosphate removal from wastewater was prepared by modifying natural wood through a top−down approach to obtain nanowood, followed by grafting polymerization to produce a porous and amino−rich cross−linked nanowood−based material (PEI−PW), and ultimately loading the Zr component on the nanowood matrix skeleton via the chelating effect of amino groups to prepare the Zr−loaded nanocomposite (PEI−PW@Zr). The adsorbent was subjected to comprehensive characterization to evaluate its potential for phosphate adsorption. To optimize the PEI−PW@Zr formulation (Zr concentration) and investigate the effects of adsorbent dosage, adsorption time, temperature, pH of the solution, and the presence of coexisting anions on the adsorption efficiency of PEI−PW@Zr for phosphate, a series of batch experiments were performed. Additionally, the cyclic regeneration performance of the adsorbent was examined.

## 2. Materials and Methods

### 2.1. Materials

Basswood was purchased from Decci Co., Ltd.: Dongguan, China. Polyethyleneimine (PEI, M. W. 70,000, 50 wt% in water), acetic acid (98 wt%), NaClO_2_ (80 wt%), ZrOCl_2_·8H_2_O (98 wt%), KH_2_PO_4_ (99 wt%), and γ−(2, 3−epoxy−propoxy) propyl trimethoxy−silane (KH560) were purchased from Aladdin Reagent Co., Ltd.: Ontario, CA, USA. NaClO was purchased from Tianjin Baishi Chemical Industry Co., Ltd.: Tianjin, China. Deionized (DI) water was made in a laboratory using an ultrapure water system (Milli−Q, Merck KGaA, Germany: Darmstadt, Germany). The porous nanowood (PW) was obtained from the laboratory based on our previous work [32,33].

### 2.2. Preparation of PEI−PW

A certain amount of PW was put into a 250 mL beaker containing 200 mL of DI water, and then 2 g of KH560 was added and magnetically stirred for 2 h. Subsequently, 12 g of PEI was added and stirred for 30 min. During the reaction, PW was squeezed continuously to accelerate the modification rate of PW. Finally, PEI−PW was obtained by washing thoroughly with DI water and freeze−drying.

### 2.3. Preparation of Zr−Loaded Adsorbent (PEI−PW@Zr)

A certain amount of dry PEI−PW was put into a 100 mL beaker, and different concentrations of ZrOCl_2_·8H_2_O aqueous solution were added to completely immerse PEI−PW. The pH was adjusted to 10 with a 0.5 M NaOH solution. Then, such solution was left at 60 °C for 2 h and at room temperature for 24 h. Finally, PEI−PW@Zr was obtained by washing thoroughly with DI water and freeze−drying.

### 2.4. Characterization

The functional groups of samples were investigated using a Nicolet iS10 Fourier−transform infrared (FTIR) spectrometer (Thermo Fisher, USA) with the wavenumber range of 4000–400 cm^−1^. The thermal behaviors of samples were performed using a TGA 550 thermogravimetric analysis (TA Instruments, USA) from 25 °C to 600 °C with a heating rate of 20 °C min^−1^ under a nitrogen atmosphere. The modification and adsorption mechanism of samples were revealed using an X-ray photoelectron spectrometer (XPS, Thermo SCIENTIFIC ESCALAB 250Xi, Waltham, MA, USA) with a monochromatic Al K alpha source operated at 230 W.

### 2.5. Adsorption Experiments

Adsorption experiments were conducted by the batch method to test the influence of adsorption kinetics, adsorption isotherm (varying from 20 to 500 mg·L^−1^) and other experiments of the concentration of ZrOCl_2_·8H_2_O (varying from 0.005 to 0.1 mol·L^−1^), the amount of adsorbent (0.5, 1, 1.5, and 2 g·L^−1^), pH (3–11), coexisting anions (SO_4_^2−^, HCO_3_^−^, and Cl^−^), and temperature (277.2, 298.2, 308.2, and 318.2 K). Before the adsorption process, the pH was adjusted to the required value by using 0.1 M NaOH and HCl. Then, a certain amount of PEI−PW@Zr adsorbent was added into a beaker containing a certain amount of phosphate solution with a known concentration and squeezed slowly at intervals. After the adsorption reached equilibrium, the phosphate concentration was measured using a UV−2550 spectrophotometer (Shimadzu, Japan). The adsorption capacity of PEI−PW@Zr was calculated by the Equation (1).

(1) $$q_{e}=\frac{\left(C_{0}-C_{e}\right)V}{m}$$

where *C*_0_ and *C_e_* are the initial phosphate concentration (mg·L^−1^) and adsorption equilibrium phosphate concentration (mg·L^−1^), respectively, *V* is the volume of the solution (L), and *m* is the mass of PEI−PW@Zr (g).

To study the stability of PEI−PW@Zr, a reusability experiment was conducted. An amount of 100 mg PEI−PW@Zr was added to 100 mL 100 mg·L^−1^ phosphate solution until reaching the adsorption equilibrium. After the adsorption, the adsorbent was taken out and regenerated with 1 wt% NaCl solution by stirring for 1 h. Finally, the adsorbent was fully rinsed with DI water to remove the residual NaCl solution, and then regenerated PEI−PW@Zr could be obtained for adsorption in succeeding cycles.

## 3. Results

### 3.1. Characterizations

**Figure 1:**
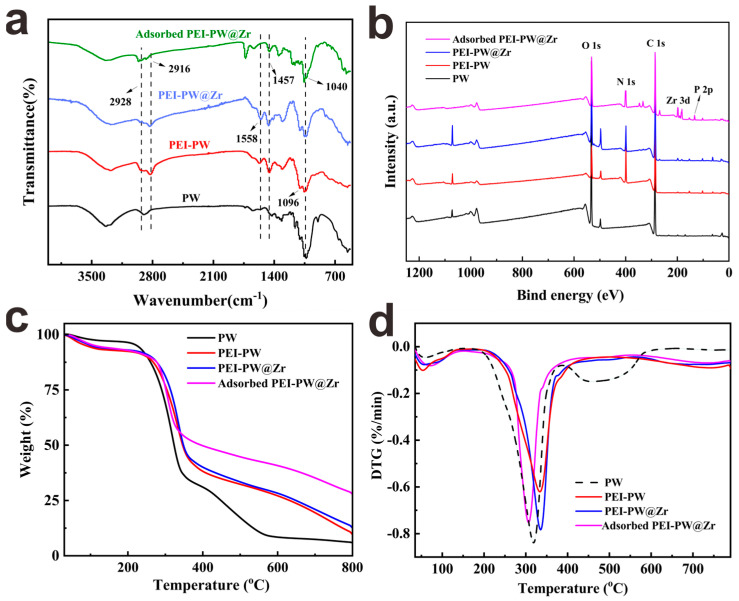
(**a**) FTIR spectra, (**b**) XPS full−scan spectra, (**c**) TGA curves, and (**d**) DTG curves of PW, PEI−PW, PEI−PW@Zr, and phosphate−adsorbed PEI−PW@Zr.

Figure 1a shows the FTIR spectra of PW, PEI−PW, PEI−PW@Zr, and phosphate−adsorbed PEI−PW@Zr. The peak at 3321 cm^−1^, attributed to the stretching vibration of O−H and N−H groups, became wider and more intense in PEI−PW compared to PW. This was due to the overlapping of stretching vibration peaks of the two groups. Additionally, two new peaks at 1558 and 1457 cm^−1^, corresponding to the stretching vibrations of C=N and N−H groups, respectively, appeared in PEI−PW [34]. The peak at 1096 cm^−1^ indicated the presence of Si−O bonds in PEI−PW. Thus, the amino group in PEI−PW was introduced through the cross−linking reaction between PEI and KH560. Moreover, the stretching vibration peaks of −CH_2_− at 2916 and 2928 cm^−1^ confirmed the successful introduction of PEI into PW. Furthermore, after phosphate adsorption, a new peak at 1040 cm^−1^ appeared in the spectrum of PEI−PW@Zr, which was attributed to the asymmetric stretching vibration of P−O, indicating the successful phosphate absorption by PEI−PW@Zr.

The changes in the surface chemical structures and elements of all samples were analyzed using XPS. The XPS full−scan spectra of PW, PEI−PW, PEI−PW@Zr, and phosphate−adsorbed PEI−PW@Zr are presented in Figure 1b. The peaks with binding energies of 181.0, 286.1, 399.2, and 532.1 eV were attributed to Zr 3d, C 1s, N 1s and O 1s, respectively. Notably, the appearance of a N 1s peak on the XPS spectrum only after the coupling reaction dendrimer verified the successful introduction of PEI in PW. Additionally, the presence of a P 2p peak at 133.1 eV after adsorption confirmed the successful adsorption of PEI−PW@Zr.

To investigate the thermal stability of the samples, TGA was conducted on PW, PEI−PW, PEI−PW@Zr, and phosphate−adsorbed PEI−PW@Zr. As presented in Figure 1c, the weight loss profiles of all four samples comprised two stages. The first stage, up to 200 °C, was attributed to the elimination of residual moisture, while the second stage (200–400 °C) corresponded to the decomposition of cellulose, amylated lignin, and other organic components of wood, wherein the C−C bond between cellulose units was broken [35]. Additionally, Figure 1c indicates that the phosphate−adsorbed PEI−PW@Zr had a higher residual mass than other samples, resulting from the successful adsorption of phosphates with flame retardant properties. The TGA analysis further revealed that the material had a maximum weight loss of approximately 300 °C, with no significant thermal decomposition below 200 °C (Figure 1d). Based on the findings, it can be concluded that PEI−PW@Zr exhibited favorable thermal stability, which is of practical significance.

### 3.2. Adsorption Study

#### 3.2.1. Effect of Zr Content

**Figure 2:**
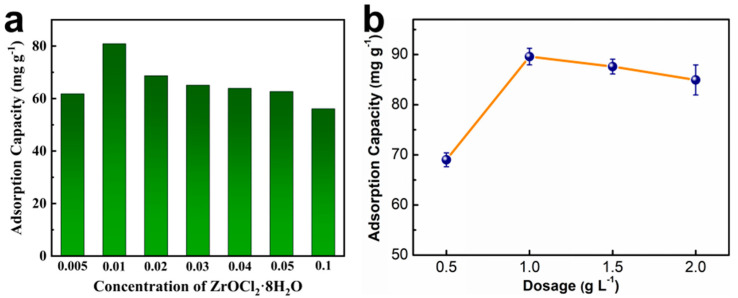
Effect of (**a**) ZrOCl_2_·8H_2_O concentration (initial phosphate concentration = 100 mg·L^−1^, adsorbent dosage = 1.0 g·L^−1^, initial pH = 3, room temperature) and (**b**) the amount of PEI−PW@Zr (initial phosphate concentration = 100 mg·L^−1^, initial pH = 3, room temperature) on adsorption capacity.

Zr content is a critical parameter that influences the adsorption performance of PEI−PW@Zr [36]. PEI−PW, which lacks active sites, exhibited minimal phosphate adsorption capacity. To investigate the effect of ZrOCl_2_·8H_2_O concentration, Figure 2a shows the phosphate adsorption by PEI−PW@Zr at various concentrations. The highest adsorption capacity (80.9 mg·g^−1^) of PEI−PW@Zr was observed at a ZrOCl_2_·8H_2_O concentration of 0.01 mol·L^−1^. At concentrations lower than this, less Zr was complexed with the amino group on the PEI−PW surface, leading to low adsorption capacity. Conversely, at concentrations higher than 0.01 mol·L^−1^, the adsorption capacity of PEI−PW@Zr decreased with an increase in Zr content. The formation of Zr(OH)_4_ particles increased significantly under alkaline conditions, leading to difficulty in entering the pores of PEI−PW and only chelating on the surface. These particles were too large to penetrate the porous structure of PEI−PW@Zr, reducing the active sites of phosphate adsorption, and were prone to detachment during the aqueous solution adsorption process. Therefore, PEI−PW@Zr with ZrOCl_2_·8H_2_O concentration of 0.01 mol·L^−1^ was chosen for subsequent adsorption experiments.

#### 3.2.2. Effect of the Amount of Adsorbent

As shown in Figure 2b, the adsorption capacity of PEI−PW@Zr increased remarkably as the amount of adsorbent increased from 0.5 g·L^−1^ to 1 g·L^−1^. This can be attributed to the fact that a larger contact area and more binding sites were provided for phosphate adsorption. However, when the amount of adsorbent exceeded 1 g·L^−1^, the adsorption capacity slowly decreased due to a reduction in unit adsorption capacity under a constant phosphate concentration. Therefore, for this experiment, an adsorbent dosage of 1 g·L^−1^ was chosen.

#### 3.2.3. Adsorption Kinetics Study

**Figure 3:**
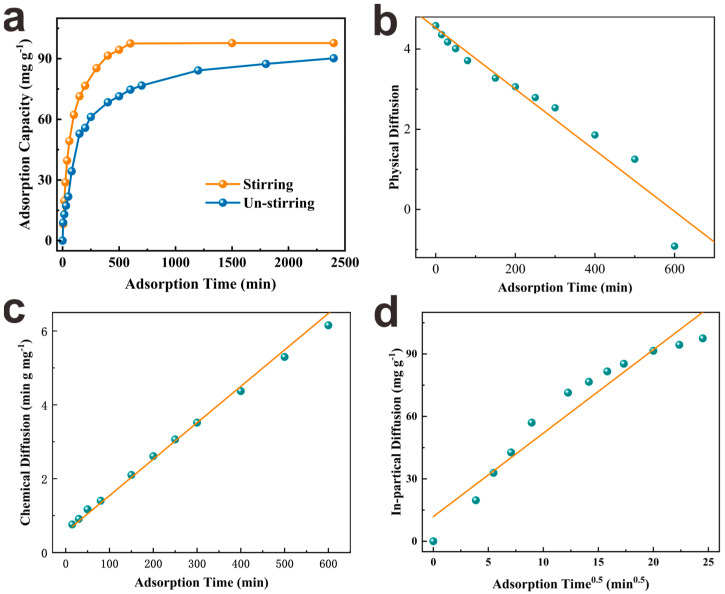
(**a**) Phosphate adsorption capacity according to contact time under stirring and un−stirring, and adsorption kinetics curve using (**b**) pseudo−first−order, (**c**) pseudo−second−order, and (**d**) intra−particle diffusion models (initial phosphate concentration = 100 mg·L^−1^, adsorbent dosage = 1.0 g·L^−1^, initial pH = 3, room temperature).

To investigate the effect of contact time between PEI−PW@Zr and phosphate on adsorption, kinetics experiments were performed and the results are presented in Figure 3a. It was observed that, when stirred, PEI−PW@Zr reached 80% of the adsorption equilibrium within 3 h and reached the adsorption equilibrium in approximately 8 h. Compared with non−stirring adsorption, the time taken for stirring adsorption to reach adsorption equilibrium decreased by 70.2%, and the adsorption capacity increased by 8.54%. The reason behind this improvement can be attributed to the large number of channels present in the as−made PEI−PW@Zr, which enhanced the mass transfer effect of phosphate liquid under stirring [37]. This increase in mass transfer improved the chances of contact and collision between phosphate and Zr on the adsorbent surface, leading to an improved adsorption rate and capacity of PEI−PW@Zr.

To elucidate the phosphate adsorption mechanism by PEI−PW@Zr, three kinetics models, namely pseudo−first−order kinetics, pseudo−second−order kinetics, and intra−particle diffusion models, were employed to fit the experimental data (see Supplementary Material) [15,28,29]. These models are primarily regulated by physical, chemical, and intra−particle diffusion processes, respectively [38]. As depicted in Figure 3b−d and Table S1, the correlation coefficient (*R*^2^) of the pseudo−second−order kinetic model was the highest among the three models, with a value of 0.9993, whereas the *R*^2^ values of the other two models were 0.9484 and 0.9352, respectively. Moreover, the equilibrium adsorption capacity of 101.32 mg·g^−1^ calculated by the pseudo−second−order kinetics model was more consistent with the actual adsorption equilibrium value (97.90 mg·g^−1^). These observations suggest that chemisorption was the dominant process during the phosphate adsorption by PEI−PW@Zr, and the pseudo−second−order kinetics model was found to be more suitable for describing the adsorption process.

#### 3.2.4. Adsorption Isotherm Study

**Figure 4:**
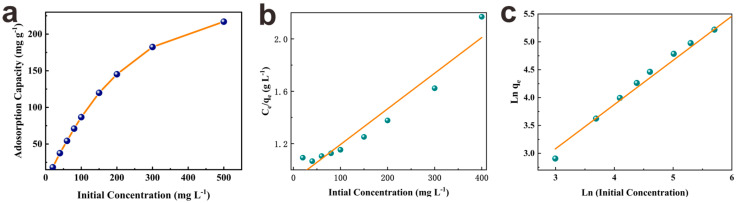
(**a**) Phosphate adsorption capacity according to initial phosphate concentration, and adsorption isotherm curve using (**b**) Langmuir model and (**c**) Freundlich model (adsorbent dosage = 1.0 g·L^−1^, initial pH = 3, room temperature).

The effect of initial phosphate concentrations on the adsorption capacity of PEI−PW@Zr and their interaction mechanism was studied through adsorption isotherm analysis, as depicted in Figure 4a. The results indicate that the adsorption capacity of PEI−PW@Zr increased markedly with an increase in phosphate concentration. As the concentration was raised to 300 mg·L^−1^ or above, the reaction sites of PEI−PW@Zr were nearly saturated, leading to a gradual flattening of the adsorption capacity. When the concentration was 500 mg·L^−1^, the adsorption capacity reached the highest, 217.0 mg·g^−1^. Moreover, the comparisons of the PEI−PW@Zr with other similar adsorbents are listed in Table S2. It can be easily seen that the adsorption capacity of PEI−PW@Zr is better than that of most similar adsorbents, which may be caused by the uniformly dispersed Zr(OH)_4_ on the wood surface.

The present study investigated the applicability of Langmuir and Freundlich isotherm models (see Supplementary Material) for describing the phosphate adsorption process of PEI−PW@Zr [4], as depicted in Figure 4b,c and Table S3. The Langmuir model assumes an ideal adsorption system with a limited number of equivalent adsorption sites distributed uniformly on the adsorbent surface, while the Freundlich model is appropriate for multilayer adsorption with a heterogeneous surface as it describes the heterogeneity of the adsorption surface [21]. Our findings showed that the *R*^2^ value of the Langmuir adsorption model was significantly higher than that of the Freundlich model. Furthermore, the maximum adsorption capacity predicted by the Langmuir adsorption model was in closer agreement with the actual adsorption equilibrium capacity of 217.0 mg·g^−1^. Hence, the Langmuir adsorption isotherm model was deemed more suitable for characterizing the phosphate adsorption process of PEI−PW@Zr, wherein the adsorption mainly occurred via monolayer adsorption and the adsorption sites on the adsorbent were evenly distributed over its surface.

#### 3.2.5. Adsorption Thermodynamic Study

**Figure 5:**
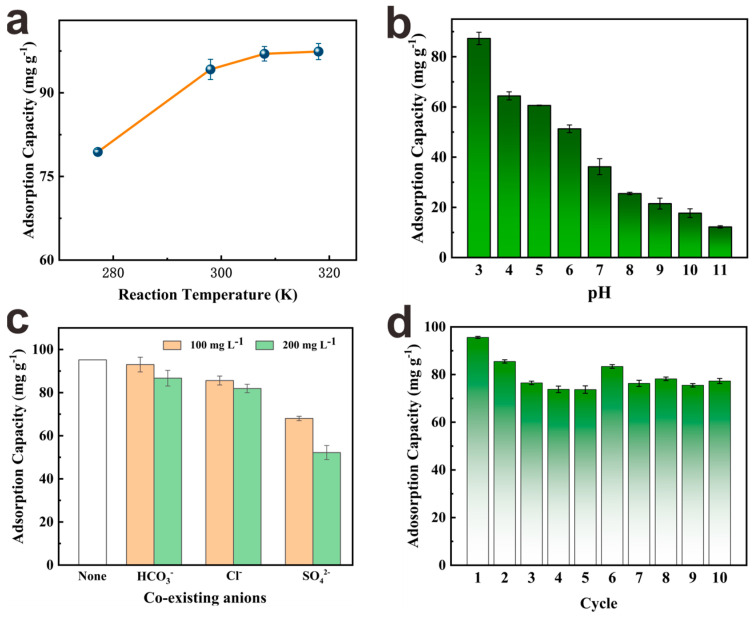
Phosphate adsorption capacity according to (**a**) reaction temperature (initial phosphate concentration = 100 mg·L^−1^, adsorbent dosage = 1.0 g·L^−1^, initial pH = 3), (**b**) pH (initial phosphate concentration = 100 mg·L^−1^, adsorbent dosage = 1.0 g·L^−1^, room temperature), and (**c**) co−existing anions (initial phosphate concentration = 100 mg·L^−1^, adsorbent dosage = 1.0 g·L^−1^, initial pH = 3, room temperature). (**d**) Phosphate adsorption by PEI−PW@Zr over 10 successive adsorption–desorption cycles (initial phosphate concentration = 100 mg·L^−1^, adsorbent dosage = 1.0 g·L^−1^, initial pH = 3, room temperature).

To gain a comprehensive understanding of the adsorption mechanism, thermodynamic parameters such as adsorption entropy (Δ*S*), adsorption enthalpy (Δ*H*), and Gibbs free energy (Δ*G*) were calculated using the van’t Hoff equation (see Supplementary Materials) [5]. The adsorption capacity of phosphate by PEI−PW@Zr at various temperatures was presented in Figure 5a and Table S4. The results show an increase in adsorption capacity with an increase in temperature, which could be attributed to the enhancement in phosphate diffusion in solution, leading to an improved binding to the active sites on the surface of the adsorbents. The calculated value of Δ*H* > 0 indicates that the adsorption process was endothermic in nature. Furthermore, the positive value of Δ*S* indicates that PEI−PW@Zr increased interfacial freedom during the adsorption process, and the negative Δ*G* values demonstrate the spontaneity of the adsorption process.

#### 3.2.6. Effect of Solution pH

The solution pH is a crucial factor affecting the adsorption process since it affects both the potential of the adsorbent surface and the form of the adsorbate [39]. As depicted in Figure 5b, the maximum adsorption capacity of 87.3 mg·g^−1^ was attained by PEI−PW@Zr at pH 3, which gradually declined with an increase in pH value. This trend could be attributed to the protonation of the amino group on PEI−PW@Zr, which imparted a positive charge to the adsorbent surface, thereby facilitating electrostatic attraction between the positively charged adsorbent and negatively charged phosphate ions under acidic conditions. As the pH value increases, the amino group deprotonates, exposing the lone electron pairs, and the adsorbent surface becomes negatively charged. Consequently, the electrostatic repulsion of phosphate ions increases, which makes it difficult for them to approach the active sites of the adsorbent, thereby causing a decline in the adsorption capacity.

#### 3.2.7. Effect of Co−Existing Anions

It is widely recognized that numerous harmful anions coexist in both industrial and domestic wastewater, which may influence the practical adsorption of phosphate by the adsorbent [40]. To investigate the effect of coexisting anions on the phosphate adsorption process, two different concentrations (100 and 200 mg·L^−1^) of competing ions were selected to coexist with phosphate for adsorption experiments, and the results are presented in Figure 5c. The inclusion of competing anions into the phosphate solution evidently affected the phosphate adsorption of PEI−PW@Zr, leading to a reduction in its adsorption capacity. As the concentration of coexisting anions increased, the adsorption capacity of the adsorbent for phosphate decreased, and the order of the effect on the adsorbent was SO_4_^2−^ > Cl^−^ > HCO_3_^−^. The observed results could be attributed to differences in the number of negative charges carried by the coexisting anions. Although the coexistence of anions affected the adsorption of phosphate by the adsorbent, it was generally within an acceptable range.

#### 3.2.8. Reusability

The reusability of adsorbents is a crucial factor in their practical application [41]. To evaluate the reusability of PEI−PW@Zr as an adsorbent for phosphate, 1 wt% NaCl solution was used as the desorption agent. Figure 5d shows that after 10 cycles, the adsorption capacity of PEI−PW@Zr decreased from 95.6 mg·g^−1^ to 77.3 mg·g^−1^, indicating that it exhibited excellent reusability. However, this reduction could be attributed to the existence of some phosphates that were tightly bound to the adsorbent and could not be completely removed during desorption. Interestingly, after the sixth adsorption–desorption cycle, the adsorption capacity of PEI−PW@Zr slightly increased, possibly due to the loosening of the adsorbent structure after prolonged soaking in water, which exposed some previously hidden adsorption sites. Nevertheless, the adsorption capacity of the adsorbent remained stable and did not decline in subsequent cycles, suggesting that the number of irreversible binding sites did not increase. Overall, the as−prepared PEI−PW@Zr exhibited good reusability for phosphate in water.

#### 3.2.9. Adsorption Mechanisms Analysis

In this work, the successful adsorption of phosphate on PEI−PW@Zr could be confirmed directly by FTIR, XPS, and TGA analyses. The phosphate adsorption by PEI−PW@Zr mainly includes two adsorption mechanisms: (1) Electrostatic attraction: According to the FTIR and XPS spectra of the samples, amino groups and Zr components have been successfully introduced into the porous wood. Thus, under the acidic conditions, the residual amino groups could be protonated to positive charge to attract the phosphate anions. (2) Ligand exchange: Due to the strong affinity of Zr for phosphate, phosphate in aqueous solution binded to Zr in the as−prepared PEI−PW@Zr through ligand exchange, thereby completing adsorption.

## 4. Conclusions

In conclusion, a phosphate adsorbent (PEI−PW@Zr) with porosity, environmental protection, and high efficiency was successfully prepared using natural wood as raw material by delignification, crosslinking, and complexing reactions. The adsorption mechanism of the adsorbent was based on electrostatic attraction and ligand exchange processes. The results of this study demonstrate that PEI−PW@Zr exhibited a high adsorption capacity (217.0 mg·g^−1^), and its adsorption kinetics and isotherm models were well described by the pseudo−second−order kinetics model and Langmuir isotherm model, respectively. In comparison with other powder adsorbents, PEI−PW@Zr exhibited a significantly improved adsorption efficiency due to its bulk structure. Moreover, PEI−PW@Zr demonstrated promising reusability with a retained adsorption capacity of over 80% after 10 regeneration cycles. Hence, the developed PEI−PW@Zr has a potential for effective removal of excess phosphate from wastewater.

## Data Availability

Data are contained within the article.

## Supplementary Materials

The following supporting information can be downloaded at https://www.mdpi.com/article/10.3390/nano13111807/s1, Figure S1: The structures of orthophosphate, pyrophosphate, long−chain polyphosphate, and phosphodiester. Table S1: Kinetic parameters of the phosphate adsorption on PEI−PW@Zr. Table S2. Comparative studies of phosphate adsorption capacity of PEI−PW@Zr and other similar adsorbents. Table S3: The fitting parameters of Langmuir and Fruendlich models of the phosphate adsorption on PEI−PW@Zr. Table S4: Thermodynamic parameters of the phosphate adsorption on PEI−PW@Zr. References [42,43,44,45,46,47,48,49] are cited in the Supplementary Materials.
