# Supplementary material for: Zirconium Component Modified Porous Nanowood for Efficient Removal of Phosphate from Aqueous Solutions
Source: Nanomaterials (Basel). 2023 Jun 5;13(11):1807. doi: 10.3390/nano13111807 (PMC10254632; doi:10.3390/nano13111807)
Supplement: Supplementary file 1 [file nanomaterials-13-01807-s001.zip › nanomaterials-2422888-supplementary.pdf]

nanomaterials

Supplementary Material

Zirconium Component Modified Porous Nanowood for Efficient Removal of Phosphate from Aqueous Solutions

Zhuangzhuang Chu [1,2,†], Wei Wang [1,†], Mengping Yin [1] and Zhuohong Yang [1,*]

[1] Key Laboratory for Biobased Materials and Energy of Ministry of Education, College of Materials and Energy, South China Agricultural University, Guangzhou 510642, China; zhuangc@scau.edu.cn (Z.C.); 18186614451@163.com (W.W.); sadheud@163.com (M.Y.)
[2] College of Natural Resources and Environment, South China Agricultural University, Guangzhou 510642, China
* Correspondence: yangzhuohong@scau.edu.cn
† These authors contributed equally to this work.

The corresponding linear fitting equations of the pseudo-first-order, pseudo-second-order and intra-particle diffusion models were listed as following equations:

The pseudo-first-order: $$\text{Ln}(q_e - q_t) = Lnq_e - k_1 t \quad \text{(S1)}$$

The pseudo-second-order: $$\frac{t}{q_t} = \frac{1}{k_2 q_e^2} + \frac{t}{q_e} \quad \text{(S2)}$$

Intra-particle diffusion: $$q_t = k_p t^{0.5} + C \quad \text{(S3)}$$

where, q_e (mg g^{-1}) and q_t (mg g^{-1}) are adsorption capacities at equilibrium and at time t, respectively; k_1 (min^{-1}), k_2 (g mg^{-1} min^{-1}) and k_p (mg g^{-1} min$^{-0.5}$) are the rate constants of pseudo-first-order, pseudo-second-order and intra-particle diffusion adsorption, respectively; C is the constant related to the thickness and boundary layer. All the kinetic parameters based pseudo-first-order, pseudo-second-order and intra-particle diffusion models can be calculated from the plots of Ln (q_e-q_t) versus t, t/q_t versus t and q_t versus $t^{0.5}$, respectively.

The linear fitting equations of the Langmuir and Freundlich models were expressed as follow:

Langmuir: $$\frac{C_e}{q_e} = \frac{1}{bq_m} + \frac{C_e}{q_m} \quad \text{(S4)}$$

Freundlich: $$\text{Ln}q_e = \frac{1}{n}\text{Ln}C_e + \text{Ln}K_F \quad \text{(S5)}$$

where, q_e (mg g^{-1}) and q_m (mg g^{-1}) are adsorption capacities of equilibrium and maximum adsorption to phosphate solution, respectively; C_e (mg L^{-1}) is the phosphate concentration at adsorption equilibrium; b (L mg^{-1}) is the Langmuir isotherm constant related to the relationship between the adsorbent and adsorbate; K_F (L g^{-1}) and n are the constants defining adsorption capacity and adsorption intensity, respectively. All the isotherm parameters based Langmuir and Freundlich isotherm models can be calculated from the plots of C_e/q_e versus $1/q_m$ and Ln q_e versus C_e, respectively.

The thermodynamics parameters for the adsorption process were calculated using the van't Hoff equation:

$$\mathrm{Ln}\frac{q_e}{C_e} = \frac{\Delta S}{R} - \frac{\Delta H}{RT} \tag{S6}$$

$$\Delta G = \Delta H - T\Delta S \tag{S7}$$

where ΔS is the entropy change (J mol^{-1} K^{-1}), ΔH is the enthalpy change (kJ mol^{-1}), R is the universal gas constant (8.314 J mol^{-1} K^{-1}), T is the reaction temperature in Kelvin (K) and ΔG is the Gibbs free energy change (kJ mol^{-1}). The values of ΔS and ΔH are calculated from the intercept and slope of linear regression of Ln q_e/C_e versus $1/T$.

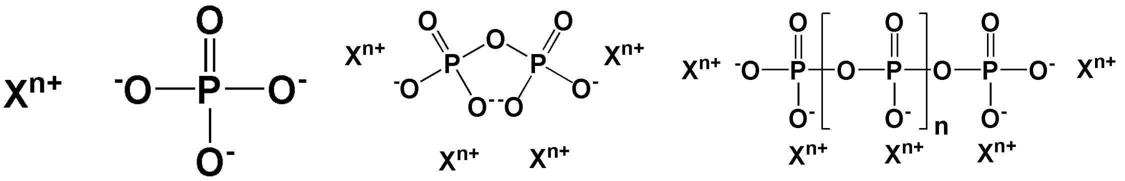

Orthophosphate Pyrophosphate Long-chain polyphosphate

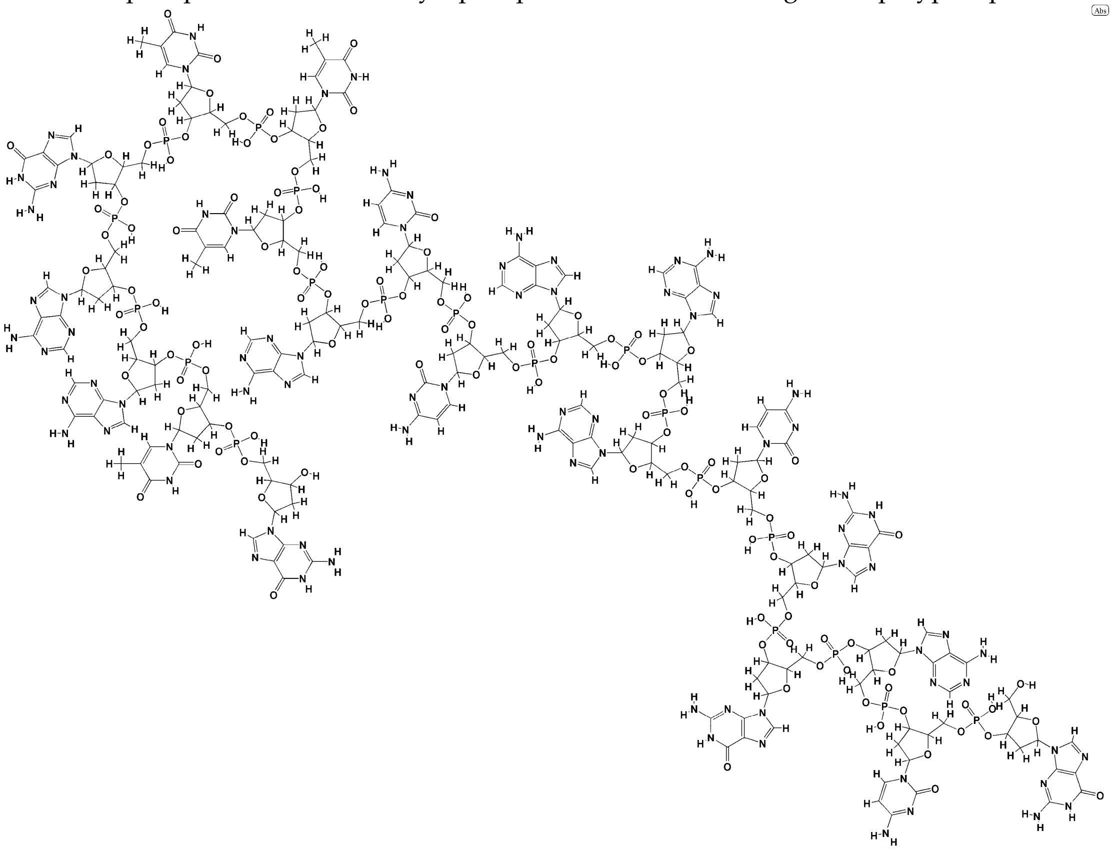

Phosphodiester

Figure S1. The structures of orthophosphate, pyrophosphate, long-chain polyphosphate, and phosphodiester.

Table S1. Kinetic parameters of the phosphate adsorption on PEI-PW@Zr.

**Adsorbent**		**PEI-PW@Zr**
$q_{e,exp}$ (mg g^{-1})		97.90
Pseudo-first-order model	q_1 (mg g^{-1})	92.61
	k_1 (min^{-1})	0.00763
	R^2	0.9484
Pseudo-second-order model	q_2 (mg g^{-1})	101.32
	k_2 (g mg^{-1} min^{-1})	1.756×10^{-4}
	R^2	0.9993
Intra-particle diffusion model	C	11.843
	k_p (mg g^{-1} min$^{-0.5}$)	4.009
	R^2	0.9352

Table S2. Comparative studies of phosphate adsorption capacity of PEI-PW@Zr and other similar adsorbents.

**Adsorbents**	**Adsorption capacity (mg g^{-1})**	**Ref.**
PEI-PW@Zr	217.0	*This work*
Zr^{4+} embedded chitosan-soya bean husk	131.3	[1]
AL-DETA@Zr	167.7	[2]
ZrCNTs	10.9	[3]
Zr@AlgKN	37.18	[4]
SP-Zr-La	61.5	[5]
Zirconium-modified zeolite	18.26	[6]
LaMOF	173.8	[7]
LaCMNF	212.0	[8]

Table S3. The fitting parameters of Langmuir and Fruendlich models of the phosphate adsorption on PEI-PW@Zr.

**Adsorbent**		**PEI-PW@Zr**
$q_{e,exp}$ (mg g^{-1})		217.0
Langmuir model	q_1 (mg g^{-1})	237.2
	b (L mg^{-1})	2.979×10^{-3}
	R^2	0.9841
Fruendlich model	KF (mg g^{-1})	3.059
	n	1.405
	R^2	0.9746

Table S4. Thermodynamic parameters of the phosphate adsorption on PEI-PW@Zr.

**ΔG (kJ mol^{-1})**				**ΔH (kJ mol^{-1})**	**ΔS (J mol^{-1} K^{-1})**
277.2 K	298.2 K	308.2 K	318.2 K	3.652	11.36
−3.145	−3.384	−3.498	−3.611		

References

1. Banu, H.T.; Karthikeyan, P.; Meenakshi, S. Zr^{4+} ions embedded chitosan-soya bean husk activated bio-char composite beads for the recovery of nitrate and phosphate ions from aqueous solution. *Int. J. Biol. Macromol.* **2019**, *130*, 573–583, https://doi.org/10.1016/j.ijbiomac.2019.02.100.
2. Zhao, Y.; Shan, X.; An, Q.; Xiao, Z.; Zhai, S. Interfacial integration of zirconium compo-nents with amino-modified lignin for selective and efficient phosphate capture. *Chem. Eng. J.* **2020**, *398*, 125561.
3. Gu, Y.; Yang, M.; Wang, W.; Han, R. Phosphate adsorption from solution by zirconi-um-loaded carbon nanotubes in batch mode. *J. Chem. Eng. Data* **2019**, *64*, 2849–2858.
4. Aswin Kumar, I.; Viswanathan, N. Fabrication of zirconium(iv) cross-linked algi-nate/kaolin hybrid beads for nitrate and phosphate retention. *Arab. J. Chem.* **2020**, *13*, 4111–4125.
5. Du, W.; Li, Y.; Xu, X.; Shang, Y.; Gao, B.; Yue, Q. Selective removal of phosphate by dual Zr and La hydroxide/cellulose-based bio-composites. *J. Colloid Interface Sci.* **2018**, *533*, 692–699, https://doi.org/10.1016/j.jcis.2018.09.002.
6. Lin, J.; Zhang, Z.; Zhan, Y. Effect of humic acid preloading on phosphate adsorption onto zirconium-modified zeolite. *Environ. Sci. Pollut. Res.* **2017**, *24*, 12195–12211, https://doi.org/10.1007/s11356-017-8873-0.
7. Zhang, X.; Sun, F.; He, J.; Xu, H.; Cui, F.; Wang, W. Robust phosphate capture over inor-ganic adsorbents derived from lanthanum metal organic frameworks. *Chem. Eng. J.* **2017**, *326*, 1086–1094.
8. Park, Y.; Gorman, C.; Ford, E. Lanthanum carbonate nanofibers for phosphorus removal from water. *J. Mater. Sci.* **2020**, *55*, 5008–5020, https://doi.org/10.1007/s10853-019-04324-8.
